# Pediatric Sleep Apnea: The Overnight Electroencephalogram as a Phenotypic Biomarker

**DOI:** 10.3389/fnins.2021.644697

**Published:** 2021-11-03

**Authors:** Gonzalo C. Gutiérrez-Tobal, Javier Gomez-Pilar, Leila Kheirandish-Gozal, Adrián Martín-Montero, Jesús Poza, Daniel Álvarez, Félix del Campo, David Gozal, Roberto Hornero

**Affiliations:** ^1^Biomedical Engineering Group, University of Valladolid, Valladolid, Spain; ^2^Centro de Investigación Biomédica en Red en Bioingeniería, Biomateriales y Nanomedicina (CIBER-BBN), Valladolid, Spain; ^3^Department of Child Health, Child Health Research Institute, The University of Missouri School of Medicine, Columbia, MO, United States; ^4^Pneumology Service, Río Hortega University Hospital, Valladolid, Spain

**Keywords:** sleep apnea, pediatrics, electroencephalography, cognition, correlation networks

## Abstract

Pediatric obstructive sleep apnea (OSA) is a prevalent disorder that disrupts sleep and is associated with neurocognitive and behavioral negative consequences, potentially hampering the development of children for years. However, its relationships with sleep electroencephalogram (EEG) have been scarcely investigated. Here, our main objective was to characterize the overnight EEG of OSA-affected children and its putative relationships with polysomnographic measures and cognitive functions. A two-step analysis involving 294 children (176 controls, 57% males, age range: 5–9 years) was conducted for this purpose. First, the activity and irregularity of overnight EEG spectrum were characterized in the typical frequency bands by means of relative spectral power and spectral entropy, respectively: δ_1_ (0.1–2 Hz), δ_2_ (2–4 Hz), θ (4–8 Hz), α (8–13 Hz), σ (10–16 Hz), β_1_ (13–19 Hz), β_2_ (19–30 Hz), and γ (30–70 Hz). Then, a correlation network analysis was conducted to evaluate relationships between them, six polysomnography variables (apnea–hypopnea index, respiratory arousal index, spontaneous arousal index, overnight minimum blood oxygen saturation, wake time after sleep onset, and sleep efficiency), and six cognitive scores (differential ability scales, Peabody picture vocabulary test, expressive vocabulary test, design copying, phonological processing, and tower test). We found that as the severity of the disease increases, OSA broadly affects sleep EEG to the point that the information from the different frequency bands becomes more similar, regardless of activity or irregularity. EEG activity and irregularity information from the most severely affected children were significantly associated with polysomnographic variables, which were coherent with both micro and macro sleep disruptions. We hypothesize that the EEG changes caused by OSA could be related to the occurrence of respiratory-related arousals, as well as thalamic inhibition in the slow oscillation generation due to increases in arousal levels aimed at recovery from respiratory events. Furthermore, relationships between sleep EEG and cognitive scores emerged regarding language, visual–spatial processing, and executive function with pronounced associations found with EEG irregularity in δ_1_ (Peabody picture vocabulary test and expressive vocabulary test maximum absolute correlations 0.61 and 0.54) and β_2_ (phonological processing, 0.74; design copying, 0.65; and Tow 0.52). Our results show that overnight EEG informs both sleep alterations and cognitive effects of pediatric OSA. Moreover, EEG irregularity provides new information that complements and expands the classic EEG activity analysis. These findings lay the foundation for the use of sleep EEG to assess cognitive changes in pediatric OSA.

## Introduction

Pediatric obstructive sleep apnea (OSA) is not only prevalent among children but also carries a significant risk for long-term morbidities primarily affecting cognitive and behavioral functioning, as well as inducing cardiovascular and metabolic dysfunction (Marcus et al., 2012). OSA-induced night time perturbations such as intermittent hypoxia, hypercapnia, and sleep fragmentation are often accompanied by systemic inflammation and oxidative stress, the latter being implicated in the neurocognitive and behavioral deficits that could hamper their intellectual and emotional development (Marcus et al., 2012; Hunter et al., 2016). Cognitive impairments have indeed been recognized as one of the major morbidities of OSA during childhood, with the most severe patients showing a higher risk of being affected (Hunter et al., 2016). Nevertheless, cognitive testing is not routinely administered to children being clinically evaluated for suspected OSA. Adenotonsillectomy has shown the reversibility of cognitive deficits associated with OSA, as well as improvements in academic results (Gozal, 1998), with suggested neurocognitive enhancements even in mild patients receiving timely treatment (Tan et al., 2017). Hence, objective identification of cognitive impairments in OSA-affected children is of paramount importance to minimize their impact and maximize their reversibility.

Sleep EEG has shown the potential to provide physiologically based cognitive information (Weichard et al., 2016; Brockmann et al., 2018, 2020; Christiansz et al., 2018) that would obviate the need for traditional neurocognitive tests, yet secure an estimate of risk for OSA-associated morbidities. However, all previous studies exploring sleep EEG and cognition focused on very specific EEG attributes, such as spindles or delta activity (Weichard et al., 2016; Brockmann et al., 2018, 2020; Christiansz et al., 2018). Consequently, how OSA alters the overnight electrical behavior of the brain of children, and whether such alterations indicate cognitive deficits, remains unclear. If such were the case, however, the intrinsic informative value of the PSG-derived EEG recordings would add further incentive to the use of PSG since it would provide not only the necessary respiratory information required for clinical treatment decision making but would also provide inferences as to the cognitive susceptibility of the patients, i.e., would enable more personalized approaches. We, therefore, hypothesized that pediatric OSA and its cognitive implications are reflected in a differential behavior of the overnight EEG. Furthermore, the recurrent nature of apneic events suggests an examination in the frequency domain. Accordingly, our main objective was to characterize new relationships between the information obtained from the overnight EEG spectrum, pediatric OSA-related polysomnographic perturbations, and cognitive functions.

To this effect, we extracted information from the conventional spectral bands of 294 EEG recordings from children, not only using the activity-based classic approach (relative spectral power, *RP*) but also the analysis of their irregularity (spectral entropy, *SpecEn*). Connections between these complementary analyses, applied to eight EEG channels, six polysomnographic variables, and six cognitive scores, were assessed using correlation networks, as they allow for an easy visualization of relationships in high-dimensional data and have been successfully used in the study of different pathological conditions (Liu et al., 2009; Barabási et al., 2011; Epskamp et al., 2012; Kwapiszewska et al., 2018; Jimeno et al., 2020). Our analytical approach is expected to identify how EEG activity and irregularity evolve as pediatric OSA worsens, while concurrently assessing their interrelationship with sleep variables and cognitive outcomes.

## Materials and Methods

### Pediatric Cohort and Sleep Studies

Community nonreferral children (169 boys/125 girls, 5–9 years old) were recruited in Chicago, Illinois, after obtaining an informed consent from their parents or legal caregivers in accordance with the Declaration of Helsinki. The protocol was approved by the Ethics Committee of the University of Chicago (protocol # 09-115-B). Polysomnography (PSG) was conducted using commercial digital equipment and scored according to the recommendations of the American Academy of Sleep Medicine (AASM) (Grigg-Damberger et al., 2007; Iber et al., 2007; Berry et al., 2012). The apnea–hypopnea index (AHI) from PSG was used as the OSA diagnostic standard. AHI common cutoffs were used to split the cohort in three subgroups: controls (AHI ≤ 1 event/h, *N* = 176), mild OSA (1 e/h ≤ AHI ≤ 5 e/h, *N* = 98), and moderate/severe OSA (5 e/h ≤ AHI, *N* = 20). Children were recruited from the sleep clinic and the pediatric otolaryngology clinics as well as by flyers posted in the community. Those children who had genetic or craniofacial syndromes and chronic diseases such as cardiac disease, diabetes, cerebral palsy, and chronic lung disease of prematurity or cystic fibrosis were excluded. In addition, any child with a known neuropsychiatric condition or developmental delay was also excluded.

### Polysomnographic Variables and Neurocognitive Tests

Six PSG-related variables were included in the study: AHI, respiratory event-related arousals (AR), minimum oxygen saturation value (Nadir_*SpO2*_), spontaneous arousals (AS), the number of minutes awake after sleep onset (WASO), and the sleep efficiency (SleepEff). AHI refers to the number of apneas and hypopneas per hour of sleep, and was used to establish the presence and severity of OSA (Berry et al., 2012). AR is the number of arousals per hour of sleep caused by abnormal respiratory events, thus, reflecting associated micro sleep disruptions. Respiratory arousals are involved in hypopnea definition, and therefore, they are also related to AHI. Nadir_*SpO2*_ is the lowest value of oxygen saturation during the night. It is very often associated with the occurrence of desaturations, which are also involved in hypopnea definition. AS is the number of spontaneous arousals. It has been included to contrast the evaluation of AR. Finally, WASO are the minutes awake after sleep onset, and SleepEff is the percentage of minutes spent asleep divided by the total of minutes in bed. Both are associated with macro sleep disruptions.

Six neurocognitive tests were administered to the children under study in the morning immediately after the PSG night (Hunter et al., 2016). Differential ability scales (DAS) is composed of a battery of subtests with ability to measure the performance of several intellectual activities of children in the range 2–17 years (Elliott, 1990b). However, in this study, it was only used as a measure of global intellectual ability by means of a composite score termed “general conceptual ability.” It merges the scores from each subtest, with a proper age standardization, showing high agreement with other common general tests (Elliott, 1990a,b). The third edition of the Peabody Picture Vocabulary Test (PPVT3) was used to assess the verbal ability of the children under study (Restrepo et al., 2006). It is a test in which children point to a picture they think that shows a word previously said aloud, i.e., it is focused on receptive verbal skills. The Expressive Vocabulary Test (EVT) is complementary to PPVT3 when evaluating language (Restrepo et al., 2006; Hunter et al., 2016). During EVT, children have to articulate the word representing the image shown in a picture, so it assesses the expressive part of language (Restrepo et al., 2006). The three remaining cognitive tests are included within NEPSY (for A Developmental NEuroPSYchological Assessment) series. Design Copying (DesCop) is intended for measuring visual–spatial processing (Ahmad and Warriner, 2001; Miller, 2007). Children are asked to copy geometrical figures, and credit is given for each partial drawing (Miller, 2007). Phonological processing (PhPro) from NEPSY assesses language in a different way than PPVT3 and EVT. While the last two refer to receptive and expressive language, respectively, PhPro measures the third subcomponent of language, called indeed phonological processing (Miller, 2007). It consists of two parts. In the first one, children have to identify words from word segments using graphic and verbal indications. In the second part, children are required to repeat a word and create a new one from the original. Finally, Tower (Tow) test is the NEPSY variant of the well-known Tower of London. It is intended for assessing executive functions, such as planning or problem solving (Baron, 2018). In less than six movements, children are asked to imitate with real pieces a given state shown in a figure (Miller, 2007).

### Signal Acquisition and Analysis

Eight EEG channels referenced to mastoids (F3, F4, C3, C4, O1, O2, T3, and T4) were acquired during PSGs at a sampling rate of 200 Hz (Grigg-Damberger et al., 2007; Iber et al., 2007). Pre-processing consisted of a four-stage methodology: (*i*) re-referencing to the average of the eight EEG channels; (*ii*) stop-band filter in 60 Hz and band-pass filter from 0.1 to 70 Hz using a Hamming window; (*iii*) automatic rejection of artifacts following an epoch-adaptive thresholding approach (Bachiller et al., 2015); and (iv) rejection of first and last parts of the EEG to avoid initial and final awake states.

The Blackman–Tukey method was used to estimate the power spectral density (PSD) of the eight EEG channels from each subject under study. A rectangular nonoverlapping window was used, with a length of 6,000 samples (30 s). The PSDs of the epochs of the whole night were averaged to estimate one PSD for each channel. Then these PSDs were normalized (PSDn) dividing its amplitude values by the total spectral power of the corresponding channel. The relative power (*RP*) and spectral entropy (*SpecEn*) of delta 1 (δ_1_: 0.1–2 Hz), delta 2 (δ_2_: 2–4 Hz), theta (θ: 4–8 Hz), alpha (α: 8–13 Hz), sigma (σ: 10–16 Hz), beta 1 (β1: 13–19 Hz), beta 2 (β_2_: 19–30 Hz), and gamma (γ: 30–70 Hz) were obtained from the PSDn of each channel (Uhlhaas and Singer, 2010). The split of delta is predicated on their different behavioral characteristics during sleep (Benoit et al., 2000), as well as in OSA presence (Gutiérrez-Tobal et al., 2019b). Sigma band was specifically analyzed because of its well-known relationship to sleep spindles (Iber et al., 2007). *RP*s were obtained per convention as it accounts for EEG activity and were computed as the sum of the PSDn amplitude values within each band:


(1) $$R\,P=\sum_{f=f\,1}^{f\,2}P\,S\,D\,n\,\left(f\right)$$

where *f*1 and *f*2 are the limits of each spectral band. *SpecEn* reflects EEG irregularity within these frequencies, regardless of total activity, thus, affording additional useful information (Inouye et al., 1991; Gutiérrez-Tobal et al., 2019a). It was obtained as follows (Inouye et al., 1991):


(2) $$S\,p\,e\,c\,E\,n=-\frac{1}{\log N}\,\sum_{f=f\,1}^{f\,2}P\,S\,D\,n\,\left(f\right)\cdot \text{log}\,\left(P\,S\,D\,n\,\left(f\right)\right)$$

which is the application of Shannon’s entropy equation to the PSDn values within *f*1 and *f*2, with *N* being the number of values within these limits. As Shannon’s entropy represents the uniformity of a given distribution, *SpecEn* quantifies the uniformity of a given spectrum in terms of its peakedness/flatness (Inouye et al., 1991). Consequently, *SpecEn* values equal to 0, the minimum in Equation (2), are reached when a single spectral component is present. This would be the case of a sinusoid in time domain, that is, a completely regular (predictable) signal. In contrast, *SpecEn* values equal to 1, the maximum in Equation (2), are reached when the power of the spectrum is equally distributed among frequencies as in the case of white noise, which is a completely irregular (unpredictable) signal in time domain (Inouye et al., 1991). According to these features, *SpecEn* should be able to characterize a redistribution of the within-band spectral power caused by OSA regardless of *RP* remaining the same in the given band.

### Correlation Network Analysis

Correlation networks are graphs based on pairwise relationships between variables (Borsboom et al., 2011). Such associations are represented as nodes—the variables—and edges—their connections—where the width and color of the later show the intensity of the correlation and its sign (in this study, red/negative and green/positive). The abovementioned six PSG outcomes and six cognitive scores were used as variables along with the activity (*RP*) and irregularity (*SpecEn*) of each EEG channel and band. A total of six correlation networks (three for *RP* and three for *SpecEn*) were dedicated to show the relationships of polysomnographic and cognitive data with the overnight EEG information in each OSA severity subgroup. Accordingly, the first step was to calculate the Spearman’s partial correlation (adjusted by sex and age) between all the variables included in the networks to form the corresponding correlation matrices. In order to cope with the different number of subjects in each OSA severity group, the correlation matrices used to estimate the networks were composed after a 1.000-run bootstrap procedure (correlation matrices with 2.5 and 97.5 percentiles are provided in the file “correlation matrices.xlsx” of the Supplementary Material). Thus, 1.000 bootstrap samples with 20 subjects from each OSA severity group were used to compute the relationship between each node of each correlation network. The subjects were randomly selected with replacement and uniform probability, and the median value of the 1.000 runs was chosen to build each network. Then, these were obtained using the R package *qgrah* (Epskamp et al., 2012). Particularly, the Fruchterman–Reingold algorithm was applied (Fruchterman and Reingold, 1991), which forced embedded network layouts after 500 iterations. Newman’s maximized algorithm was used to conduct a modularity analysis to show possible clusters in the networks (Newman, 2006; Rubinov and Sporns, 2010). It measures the degree in which a network can be divided into different related and nonoverlapping clusters and, at the same time, provides the composition of such clusters (Rubinov and Sporns, 2010), i.e., the nodes assigned to each of them. An ancillary analysis of centrality of the nodes was assessed using *strength*, *closeness*, and *betweenness* (Rubinov and Sporns, 2010), whose results can be seen as Supplementary Figures.

### Statistical Analysis

Mann–Whitney nonparametric *U*-test was used to evaluate differences between OSA severity groups in age, body mass index, clinical variables, and cognitive scores. Fisher’s exact test was conducted to evaluate these differences in sex. Spearman’s partial correlation (*ρ*), adjusted by the sex and age of children, were used in the correlation networks. R package *qgraph* was used to obtain the corresponding network graphs (Epskamp et al., 2012). Only non-negligible absolute correlation values (| *ρ*| ≥ 0.30) were shown in the correlation networks (Mukaka, 2012).

## Results

### Polysomnography Variables and Cognitive Scores

Table 1 shows the summary of the PSG variables and cognitive scores (median and interquartile range) in the 294 subjects divided according to OSA subgroups. Sociodemographic characteristics (age, sex, and body mass index) are also presented. As would be anticipated from the delineation of the groups, AHI, AR, and Nadir_*SpO2*_ showed statistically significant differences (*p*-value < 0.05, Mann–Whitney *U*-test) between them, while AS was significantly lower in moderate/severe OSA. All cognitive scores showed a decreasing tendency as OSA severity increases, with DAS, PhPro, and Tow reaching significant differences. No statistically significant differences emerged for age, sex (Fisher’s exact test), WASO, and SleepEff.

**TABLE 1:** Sociodemographic data, polysomnography (PSG) variables, and cognitive scores in the three groups.

Data	Controls (*N* = 176)	Mild obstructive sleep apnea (OSA) (*N* = 98)	Moderate/severe OSA (*N* = 20)	*p* < 0.05
Age (years)	6.92 (6.50, 7.42)	6.92 (6.50, 7.42)	6.81 (6.37, 7.29)	n.s
Sex (M/F)	104/72 (59%)	55/43 (56%)	10/10 (50%)	n.s
BMIz	0.65 (−0.11, 1.47)	0.76 (−0.14, 2.04)	1.70 (−0.08, 2.24)	^b^
AHI (e/h)	0.40 (0.10, 0.60)	1.50 (1.20, 2.20)	9.20 (7.30, 17.20)	^a, b, c^
AR (e/h)	0.30 (0.05, 0.80)	1.00 (0.40, 2.82)	7.30 (4.88, 9.55)	^a, b, c^
AS (e/h)	6.70 (4.70, 9.00)	6.60 (4.20, 9.00)	3.10 (1.52, 6.88)	^b, c^
Nadir_*SpO2*_ (%)	94.00 (92.00, 95.00)	91.00 (89.00, 94.00)	84.00 (75.00, 87.00)	^a, b, c^
WASO (min)	45.50 (27.00, 79.50)	37.50 (23.30, 64.30)	41.00 (19.80, 75.40)	n.s
SleepEff (%)	90.60 (84.03, 94.10)	91.00 (85.23, 94.50)	91.00 (85.45, 95.05)	n.s
DAS	101.50 (92.00, 111.50)	100.50 (86.00, 111.00)	97.00 (85.00, 104.00)	^b^
PPVT3	99.00 (89.50, 110.00)	98.00 (89.80, 109.30)	96.00 (88.25, 101.50)	n.s
EVT	100.00 (89.30, 108.00)	97.00 (85.50, 105.00)	96.50 (91.00, 99.00)	n.s
DesCop	11.00 (8.00, 13.00)	10.00 (7.00, 12.00)	9.00 (7.50,11.00)	n.s
PhPro	10.00 (8.00, 12.00)	9.00 (8.00, 13.00)	7.50 (5.50, 10.00)	^b, c^
Tow	12.00 (10.00, 14.00)	11.00 (9.00, 14.00)	9.50 (7.00, 11.50)	^b, c^

*AHI, apnea–hypopnea index; AR, respiratory arousal index; AS, spontaneous arousal index; BMIz, standardized body mass index; DAS, differential ability scales; DesCop, design copying; EVT, expressive vocabulary test; Nadir_*SpO2*_, overnight minimum oxygen saturation value; PhPro, phonological processing; PPVT3, Peabody picture vocabulary test; SleepEff, sleep efficiency, WASO, time awake after sleep onset; Tow, Tower test ^*a*^Controls vs. mild OSA comparison. ^*b*^Controls vs. moderate/severe OSA comparison. ^*c*^Mild OSA vs. moderate/severe OSA comparison. n.s, not significant.*

### Averaged Electroencephalogram Spectrum of the Three Obstructive Sleep Apnea Severity Categories

**FIGURE 1:**
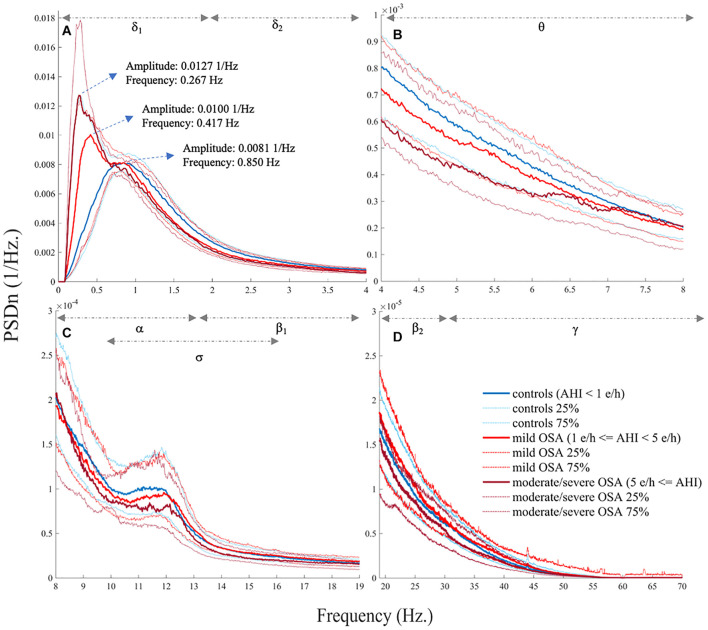
Electroencephalogram (EEG) normalized spectrum (PSDn) averaged for the three obstructive sleep apnea (OSA) severity groups (median, 25%, and 75% quartiles), showing **(A)** δ_1_ and δ_2_, **(B)** θ, **(C)** α, σ, and β_1_, and **(D)** β_2_ and γ.

Figures 1A–D show the averaged EEG PSDn’s from the three OSA severity degrees considered. First, the normalized spectrum from the eight EEG channels was averaged for each subject. Then, the median and quartile values within each OSA group were obtained for each frequency to be illustrated in the figure. As shown in Figure 1A, a peak coherent with the typical slow oscillation (SO) wave from δ_1_ gradually decreases in frequency and increases in relative power as OSA severity is higher. In addition, the spectrum from δ_2_ onward (except for α band) tends to flatten (notice the scale of the Figures 1B–D) with OSA severity, particularly when comparing controls and moderate/severe OSA.

### Overall Evolution of the Electroencephalogram Relationships With Polysomnography Variables and Cognitive Scores

**FIGURE 2:**
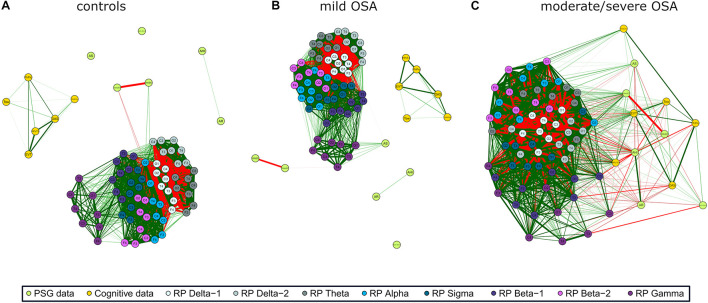
EEG activity (*RP*) correlation networks with polysomnography (PSG) variables [apnea–hypopnea index (AHI), AE, respiratory event-related arousals (AR), minimum oxygen saturation value (Nadir_*SpO2*_), the number of minutes awake after sleep onset (WASO), and sleep efficiency (SleepEff)] and cognitive scores [differential ability scales (DAS), Peabody Picture Vocabulary Test (PPVT3), Expressive Vocabulary Test (EVT), design copying (DesCop), phonological processing (PhPro), and Tower (Tow)] for **(A)** controls, **(B)** mild OSA, and **(C)** moderate/severe OSA. Wider edges are higher Spearman’s correlation absolute values | *ρ*|, with red color meaning negative correlation and green positive correlation. Solid lines represent | *ρ*| ≥ 0.50.

**FIGURE 3:**
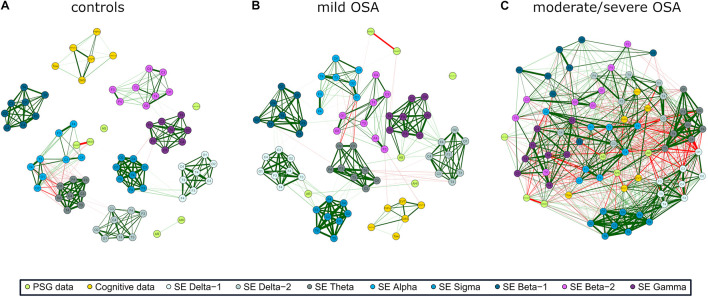
EEG irregularity (*SpecEn*) correlation networks with PSG variables (AHI, AE, AR, NadirSpO2, WASO, and SleepEff) and cognitive scores (DAS, PPVT3, EVT, DesCop, PhPro, and Tow) for **(A)** controls, **(B)** mild OSA, and **(C)** moderate/severe OSA. Explanations regarding the construction of the networks are analogous to those from Figure 2.

The three networks built using the activity of EEG channels (*RPs*) show high relationships within and between spectral bands (Figures 2A–C), i.e., a compact behavior in the activity information that reflects its similarity. However, major associations with PSG and cognitive nodes only arise for moderate/severe OSA (Figure 2C). In the corresponding irregularity (*SpecEn*) networks (Figures 3A–C), the behavior of the EEG nodes progresses from disaggregated by spectral bands (controls) to strong relationships between these (moderate/severe OSA). Similarly, only a few non-negligible relationships arise between irregularity nodes and PSG or cognitive variables in controls, but this behavior disappears as OSA worsens, reaching the maximum correlations in the moderate/severe group. Therefore, in both activity and irregularity networks, the development of OSA increases the absolute correlations between EEG and non-EEG nodes, as well as between EEG nodes from different spectral bands.

All correlation values between nodes are in the Supplementary Material (“correlation matrices.xlsx”), along with the networks corresponding to 95% confidence interval derived from the bootstrap procedure. The most relevant correlation values are also shown in the next sections.

### Spectral Band Average Associations With Polysomnography Variables and Cognitive Scores

**FIGURE 4:**
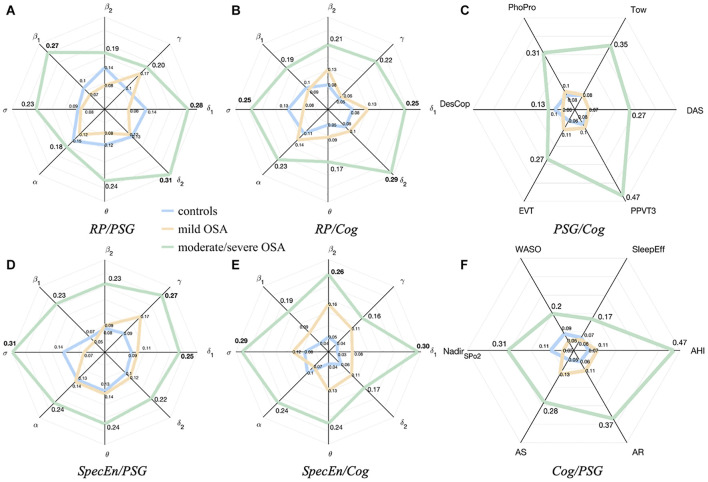
Radar charts summarizing the averaged absolute correlation values between the sleep EEG, the PSG variables, and the cognitive scores. **(A,B)** correspond to relationships with EEG activity (*RP*). **(D,E)** correspond to relationships with EEG irregularity (*SpecEn*). **(C,F)** correspond to averaged correlations of PSG variables with each cognitive score and averages cognitive scores with each PSG variable. The distances from the center represent the average absolute correlations for controls (blue), mild OSA (yellow), and moderate/severe OSA (green).

Figures 4A,B,D,E are radar (spider) charts showing channel-averaged correlations between the EEG spectral bands and the non-EEG variables. As expected, the overall tendency reflects higher absolute correlations with PSG and cognitive variables as OSA severity increases, for both EEG activity (*RP*) and irregularity (*SpecEn*). The tendency is only somehow different for the relationships between *RP* and PSG nodes (Figure 4A), where the averaged absolute correlation is generally higher in controls than in mild OSA.

Average relationships between EEG irregularity and PSG variables of mild and moderate/severe OSA are higher than the equivalent for EEG activity, as reflected by the values of the corresponding radar charts (Figures 4A,D). The highest absolute averaged correlations, reached in the moderate/severe group, are mainly influenced by EEG activity from δ_1_ (0.28), δ_2_ (0.31), and β_1_ (0.27), and EEG irregularity from δ_1_ (0.25), σ (0.31), and γ (0.27).

Relationships between EEG irregularity and activity with cognitive scores are more similar (Figures 4B,E), but still more spectral bands show higher averaged correlations in the case of *SpecEn* for both mild and moderate/severe OSA, the latter reaching the maximums values again in δ_1_ (0.25) and δ_2_ (0.29), and σ (0.25) for EEG activity, and δ_1_ (0.30), σ (0.29), and β_2_ (0.26) for EEG irregularity.

Radar charts with the relationships between PSG and cognitive variables were also generated for completeness of the analysis and are shown in Figures 4C,F. As can be observed, the relationship between PSG variables and cognitive scores also increases with OSA severity.

### Modularity Analysis and Specific Relationships

**FIGURE 5:**
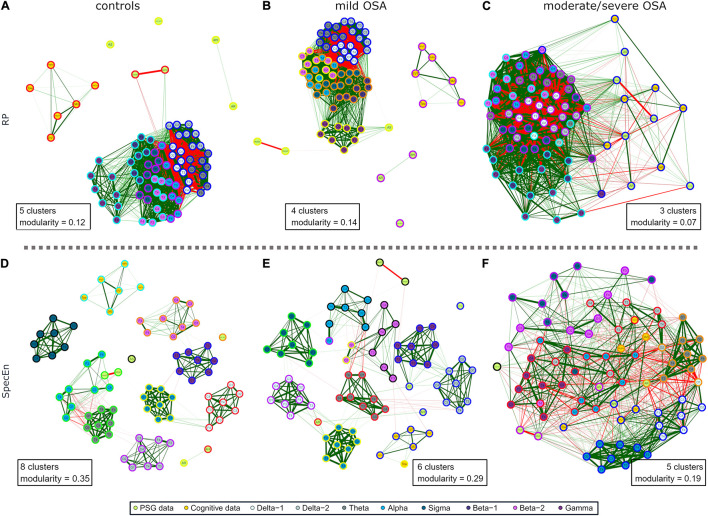
Modularity analysis of the correlation networks of each group using **(A–C)** EEG activity (*RP*) and **(D–F)** EEG irregularity (*SpecEn*). The color of the external circles represents belonging to the same module within each correlation network.

Coherent with its compact behavior, a low number of groups of especially related nodes (termed modules or clusters) were obtained after applying the modularity analysis to the EEG activity networks: five modules for controls, four for mild OSA, and three for the moderate/severe group (Figures 5A–C). This was summarized and quantified in the maximized modularity measure that reached very low values, meaning a low expected modular behavior (0.12, 0.14, and 0.07, respectively) (Newman, 2006). Node distribution through modules agrees with the evolution of the relationships of EEG activity and PSG/cognitive variables from controls to moderate/severe OSA, the latter showing a module shared by all non-EEG nodes and the C3 channels of β_2_ and γ bands.

The EEG irregularity networks showed a more modular behavior (Figures 5D–F). Controls, mild OSA, and moderate/severe OSA showed eight, six, and five modules, respectively, with concordant maximized modularity values (0.35, 0.29, and 0.19) that quantify the vanishing of the clustering tendency. OSA worsening implied modules more shared by EEG and non-EEG nodes, as well as by EEG nodes from different spectral bands because of increased absolute correlations between them. Interestingly, four out of the five modules of moderate/severe OSA (Figure 5F) are shared by both EEG and non-EEG nodes. AHI, PPVT3, EVT, and Tow are with all the σ band nodes and most of δ_1_. Similarly, AR, DAS, and PhPro are with all θ nodes and one node from δ_1_ and δ_2_. AS, WASO, and SleepEff are with most of β_1_ and β_2_. DesCop is with all α and γ nodes and most of δ_2_. Finally, Nadir_*SpO2*_ is the single node of the last module.

Supplementary Figures 1–8 show further assessment on the networks based on stability and centrality measures (Rubinov and Sporns, 2010).

## Discussion

The approaches undertaken to process the overnight EEG signal show the overall evolution of the activity (*RP*) and irregularity (*SpecEn*) of the overnight EEG as a function of pediatric OSA severity. Implicitly, we have also assessed the ability of *RP* and *SpecEn* to characterize the effects of pediatric OSA in the sleep EEG, with *SpecEn* being specifically used for the first time toward this goal. As such, current findings obtained using correlation networks unravel the existence of novel specific relationships between both activity and irregularity of the EEG, PSG variables, and cognitive scores in children with OSA. These initial observations open the door to more intense explorative analyses of the PSG as a source of not only clinical information regarding respiratory disturbance but also to provide improved phenotyping of cognitive morbidity in such patients, thereby allowing for tailored and personalized interventions and follow-up.

### Electroencephalogram Correlation Networks Evolves With Obstructive Sleep Apnea Worsening

The behavior of the networks showed higher absolute correlations between the nodes as OSA severity increased, regardless of whether these were related to PSG, cognition, or EEG. Such evolution was supported by higher network densities, decreasing number of modules, and lower maximized modularity values. These results support the idea of a gradual pathological expression of OSA in the overnight EEG spectrum, with only the step between controls and mild OSA in the activity networks slightly disagreeing with this general tendency. This *a priori* incongruence might be explained by the AHI range represented in our mild OSA group, whose median value (1.5 e/h) is much closer to control threshold (1 e/h) than to the low limit of moderate/severe OSA (5 e/h). This is a potential limitation of our study, whereby the absence of equally distributed AHI values in mild OSA may be hiding higher correlations with the EEG information. However, further investigation would be required to confirm these premises.

Activity *RP* networks were denser and less modular than irregularity *SpecEn* networks. This finding reveals the presence of more similarities among the information offered by *RP* than the corresponding one provided by *SpecEn*, suggesting the representation of a broader variety of information by the latter. However, in both activity and irregularity networks, the information contained in the overnight EEG spectrum became more similar as OSA worsened. Although this effect is clearer for irregularity spectral bands, it is also present in activity ones, which suggests that OSA affects EEG over a wide range. This finding is consistent with recent studies on continuous influence on the EEG of OSA-affected children due to different abnormal respiratory patterns during sleep (Guilleminault et al., 2019).

The moderate/severe OSA group showed the strongest correlations between EEG and non-EEG nodes. This is not surprising for the PSG variables, since they either directly reflect pathophysiological events (AHI, AR, and Nadir_*SpO2*_), or they are indirectly affected by the occurrence of these (WASO, SleepEff, and AS). Regarding cognitive scores, only EEG irregularity in β_2_ showed non-negligible (but weak) relationships in networks other than those from the moderate/severe group. The combined indication of these weak associations and the stronger correlations found for the highest severity degree, may be suggesting that sleep EEG does not robustly reflect cognition, in general, at least in an overnight scale, but reflects cognitive alterations in the presence of the most severe degrees of OSA. These results agree with the decreasing tendencies in all our cognitive scores as OSA worsens and the significantly lower values in some of these measures. Our findings also agree with previous reports pointing to a higher risk of cognitive deficits in moderate/severe OSA children (Hunter et al., 2016).

### Electroencephalogram Activity and Irregularity Characterize Specific Relationships

Correlation network and modularity analyses highlighted interesting associations of the EEG with PSG and cognitive variables in moderate/severe OSA. EEG δ_1_ and δ_2_ bands played a key role in both activity and irregularity networks. As reflected in Figures 2C, 5C, activity in these bands was mainly associated with the PSG variables AHI, SleepEff, (δ_1_ and δ_2_), and AR (δ_2_)—absolute correlation through the EEG channels in the ranges 0.35–0.58, 0.26–0.48, and 0.31–0.43, respectively, as well as the cognitive scores PPVT3, EVT, and Tow—ranging 0.30–0.53, 0.31–0.53, and 0.31–0.42. Similarly, irregularity in δ_1_ and δ_2_ was mainly related to AHI (δ_1_), AR (δ_1_ and δ_2_), and AS (δ_1_ and δ_2_), ranging 0.41–0.48, 0.31–0.66, and 0.31–0.52, respectively, with δ_1_ being also associated with DAS (0.33–0.38), PPVT3 (0.49–0.61), and EVT (0.32–0.54). EEG σ band also exhibited meaningful associations in both networks, with activity being principally related to PSG variables AS (0.19–0.48) and SleepEff (0.11–0.50) and with the cognitive score PPVT (0.31–0.49). Likewise, σ irregularity was mainly related to AHI (0.35–0.58), SleepEff (0.28–0.51), PPVT3 (0.43–0.59), and EVT (0.36–0.46).

Interestingly, EEG irregularity also showed major absolute correlations beyond δ_1_, δ_2_, and σ: with PSG variables in θ (AHI: 0.30–0.57, AR: 0.46–0.67) and γ (AHI: 0.34–0.61, WASO: 0.40–0.55), and with cognitive scores in β_2_ (DesCop: 0.31–0.65, PhPro: 0.32–0.74, Tow: 0.32–0.52). These irregularity further associations reached the strongest correlations of any single EEG node with AHI (0.61), AR (0.67), PhPro (0.74), and Tow (0.52), or were very close to the strongest for WASO (0.57) and DesCop (0.66), which were also obtained using irregularity. The EEG activity nodes, thus, reached higher maximum absolute correlations with non-EEG variables only in relationships with DAS (0.58) and Nadir_*SpO2*_ (0.60). This suggests the usefulness of irregularity measured by *SpecEn* to characterize both physiological perturbations and cognitive effects, which adds novelty to the classic activity-based analysis.

Another interesting comparison arose when assessing the maximum absolute correlations found for the PSG variables and cognitive scores in the moderate/severe OSA group. Whereas radar charts (Figure 4) showed half of the averaged correlations between PSG/Cog and Cog/PSG higher than those with EEG nodes, only AHI, PPVT3 and Tow reached the highest values in nodes other than *RP* or *SpecEn*. Accordingly, 14, 7, 3, 5, and 14 EEG nodes (either *RP* or *SpecEn*) reached absolute correlations higher than the highest with non-EEG nodes for AR, AS, Nadir_*SpO2*_, WASO, and SleepEff, respectively. Similarly, 6, 33, 32, and 1 EEG nodes reached absolute correlations higher than the highest with non-EEG nodes for DAS, EVT, DesCop, and PhPro, respectively. Moreover, the maximum absolute values for AHI (0.65 with PPVT3), PPVT3 (0.65 with AHI), and Tow (0.58 with AHI) were almost reached by EEG nodes too (0.61, 0.62, and 0.52, respectively). These figures highlight that the information contained in the EEG reaches a more complete characterization of the cognitive performance than PSG variables in moderate/severe OSA, as well as a more complete characterization of this disease state than cognitive scores. Moreover, in contrast to the PSG variables and cognitive scores, the *SpecEn* and *RP* computing is automated.

### Correlation Networks Help Expand Current Knowledge

The found relationship between δ activity and AHI agrees with previous studies reporting differences in slow wave sleep activity (SWS) in OSA-affected children (Bandla and Gozal, 2000; Christiansz et al., 2018). Moreover, we have shown that not only activity in δ but also irregularity in δ, θ, σ, and γ reflects AHI. These bands have been previously associated with arousals and other different wakefulness states (De Gennaro et al., 2001; Scholle and Zwacka, 2001; Cantero et al., 2004; Vanhatalo et al., 2004; Le Van Quyen et al., 2010), suggesting that the most consistent relationships between moderate/severe OSA and EEG activity and irregularity are related to micro and macro sleep disruptions. Coherent with this idea are the also uncovered associations between the EEG information and AR (δ_1_, δ_2_, and θ), SleepEff (δ_1_, δ_2_, θ, σ, and γ), and WASO (γ).

A few studies exist assessing relationships between δ and cognition during sleep in either healthy or OSA-affected children. Weichard et al. (2016) analyzed the EEG of 42 children (13 controls, 15 resolved OSA, and 14 unresolved OSA). They found associations between increased verbal performance and late SWS, which agrees with the relationships of EEG activity in δ_1_/δ_2_ with PPVT3 (receptive language) and EVT (expressive language) shown in this study. Interestingly, we found stronger relationships between δ_1_ and receptive and expressive language using EEG irregularity. Similarly, neither their study nor ours report strong associations of δ activity with DesCop (visual–spatial processing) and PhPro (phonological processing). However, we do expose robust associations of EEG irregularity in β_2_ with both cognitive scores. Christiansz et al. (2018) extended the previous work to 72 children and found associations between SWS activity and impaired executive function in OSA presence, showing absolute correlations of 10 different tests in the range 0.33–0.78 (Christiansz et al., 2018). Their observations agree with the non-negligible correlations found between Tow test and δ_1_/δ_2_ activity of moderate/severe OSA (0.31–0.42). Moreover, our method allowed us to find the strongest correlation with Tow score using β_2_ irregularity (0.52).

Brockmann et al. (2018) implemented a different approach by assessing the spindle pattern of 14 controls and 19 mild OSA children. Spindle density was significantly lower in the latter, which also showed associations with Wechsler Intelligence Test for Children total IQ, verbal comprehension, working memory, and processing speed (Brockmann et al., 2018). Spindle frequencies in children (≈10–16 Hz.) are within σ band (Purcell et al., 2017; Markovic et al., 2020), which showed only negligible associations in our mild OSA group. This discrepancy may be due to the different cognitive tests used and that sleep spindles occur mostly in N2 non-rapid-eye movement (NREM) sleep. However, we found some robust relationships between the cognitive scores of moderate/severe OSA with the corresponding σ activity and irregularity values (see “correlation matrices.xlsx”), thus, pointing again to the overrepresented low AHI values in our mild OSA group as the cause for the differences with their results. Brockmann et al. (2020) complemented their previous study by assessing spindle differences in 20 control children and 20 primary snorers, who showed decreased spindle density. This is an interesting finding that agrees with the decreased activity and regularity of our σ band as OSA degree is higher. However, we are precluded from further comparisons since the inclusion of a primary snoring group is both a limitation of our study and a future goal.

### Interpretations of the *SpecEn* Characterization on Sleep Electroencephalogram

A preliminary effort focused on the analysis of overnight EEG activity in the context of pediatric OSA (Gutiérrez-Tobal et al., 2019b) laid the foundation for the in-depth evaluation conducted in this study, including the use of *SpecEn*, a wider range of sleep cognitive scores, and common sleep indices from the PSG. As a result, *SpecEn* demonstrated its ability to characterize both PSG variables and cognitive scores, particularly in the case of moderate/severe OSA children, enabling higher absolute correlations than *RP* with most of the non-EEG nodes considered.

According to the correlation network and modularity analyses, the *SpecEn* ability to characterize a wider range of EEG information may underlie these improvements in the strength of the associations identified herein. One reason for such superiority as shown by *SpecEn* may be related to the finite nature of *RP*. Spectrum normalization by its total spectral power is a common tool to avoid the characterization of features different from the object of the study, which, in this case, are the OSA effects on sleep EEG. However, this technique leads to the sum of all *RP*s from the same EEG being 1, thus, providing the *RP* from each spectral band with a competitive essence. Consequently, the *RP* from one spectral band may be related to the others either because a genuine subjacent event is reflected in several spectral bands or, if this shared event does not exist, because an increase in the *RP* of one spectral band means a decrease in the *RP* in the others (to end up with a total sum of 1). This characteristic would also explain the less modular behavior of the *RP* networks. In contrast, the shape of the spectrum (its peakedness or flatness) does not impose the same limitation, since a dominant peak in one spectral band does not imply changes in the occurrence of dominant peaks in other spectral bands. Consequently, one possibility is that *SpecEn* relationships between spectral bands may be reflecting only genuine subjacent events. One example would be the positive relationships between δ_1_, σ, and γ found in the *SpecEn* correlation network of controls, which would be coherent with the hierarchical relationships between SO, spindles, and ripples described in the literature (Staresina et al., 2015). Another example in the same network would be the negative relationships found in the occipital channels of θ and α, which are coherent with the transitions between N1 and “wake” stages in which they are involved, respectively (Iber et al., 2007).

Assuming that the *SpecEn* sleep EEG characterization indeed reflects genuine subjacent events, interesting physiological interpretations can be derived from our results. First, as mentioned above, Figure 1A shows a decrease in SO frequency with OSA severity. In the control group, SO is located within its normal range: ≈0.75 Hz and within (0.55–0.95 Hz) (Achermann and Borbély, 1997). However, the frequency gradually slows down for mild OSA (0.417 Hz) and moderate/severe OSA (0.267 Hz). A progressive increase in the amplitude of the SO peak can be also observed from 0.0081 1/Hz in controls to 0.0100 1/Hz, and 0.0127 1/Hz in mild and moderate/severe OSA. SOs are sleep waves characterized by periods in which cortical and thalamic neurons alternate states of intense synaptic activity, or up states, with the almost complete absence of activity, or down states (Neske, 2016). The functions of SO are still under discussion, but growing evidence suggests that they comprise at least the synchronization of higher frequency oscillations, memory consolidation, and biochemical regulation of neurons during down states (Neske, 2016). Both cortex and thalamus are involved in SO, the latter playing key roles in generating the up state (i.e., the generation of the oscillation period) and the synchronization of faster oscillations (David et al., 2013; Neske, 2016). It has been also observed that the suppression of the thalamic role leads to a deceleration of the typical SO frequency in rodents, suggesting cortical attempts to mimic the role of thalamus (David et al., 2013; Neske, 2016). Accordingly, our results may be showing that OSA inhibits the role of thalamus in SO, with this inhibition becoming more intense as the illness is more severe. Moreover, the increased normalized power in the corresponding SO frequencies of mild and moderate/severe OSA may be reflecting that more time is spent overnight in these frequencies compared with controls. This increased time could be related to an inefficiency of the cortex when assuming the abovementioned thalamus roles. Concurrently, *SpecEn* in δ_1_ may be characterizing an increasing regular behavior of the cortex when trying to compensate the absence of the thalamus as this is more inhibited. Why thalamus function is inhibited with OSA remains unclear. However, it might be related to an increase in the consciousness/arousal degree that would be needed to recover from respiratory events. In the absence of a proper evaluation of this hypothesis, it would be supported by the fact that the cortex activity is increased, as well as by previous studies reporting that the power in δ band is higher and the EEG irregularity is lower when recovering from OSA-related respiratory events (Huang et al., 2018).

A second interpretation can be derived from the flattening experimented in most of the spectral bands beyond δ_1_, particularly in the moderate/severe OSA group. A significant increased number of respiratory arousals per hour (see Table 1) may be one possible explanation. These EEG events are known to present frequencies in the range of θ, α (except spindles), β_1_, β_2_, and γ (Iber et al., 2007). They have been also related to some changes in δ band (Bandla and Gozal, 2000; Bruce et al., 2011). Therefore, they can contribute to the spectral power of almost the whole frequency range in a white noise-like behavior. This means that adding these events to the normal EEG could make all its spectral components to be more distributed or flatter, thus, increasing the information similarity among the affected spectral bands. In our study, the meaningful correlations found in all the spectral bands between *SpecEn* and AR make respiratory arousals one of the most central nodes of the moderate/severe correlation network (see Supplementary Figure 6), thus, supporting this explanation. In addition, previous works have reported positive correlations between entropy measures on hypnogram and traditional sleep fragmentation measures such as arousal index and sleep efficiency (Kirsch et al., 2012). Another explanation, which does not exclude the previous one, is related to the abovementioned inefficiency of cortex when mimicking the role of thalamus to synchronize higher-frequency oscillations (Neske, 2016). If such synchronization is not properly conducted in moderate/severe patients, a regular behavior is lost (or at least reduced), thus, increasing the EEG irregularity and, consequently, the flatness of the affected spectral components. Further *ad hoc* studies would be required to assess whether any of these two explanations are right.

Finally, to complete the *SpecEn* interpretation, we propose a connection with our cognitive results. A recent systematic review has established speech and language problems in children suffering from OSA (Mohammed et al., 2021). This is aligned with the maximum correlations found between *SpecEn* in δ_1_ and PPVT3 (+0.61) and EVT (+0.54) of moderate/severe OSA children, which could be indicating that the impairment of these verbal skills could be measured through the increased regularity (increased peakedness) in this spectral band. Accordingly, the language problems could be somehow associated with the abovementioned thalamus inhibition in SO due to OSA. Interestingly, the third language ability score evaluated in this study, PhPro, was strongly associated with irregularity in β_2_ (+0.74), suggesting a different physiological process involved. This idea would be supported by the other correlations found between β_2_ and DesCop (+0.65) and Tow (+0.52) scores, which account for visuospatial processing and executive function, respectively. Beta oscillations are common in REM sleep (Vijayan et al., 2017). Although the role of REM sleep and cognition has not been completely delineated, it has been linked to neural network reorganization leading to new neural associations and an increased creativity (Cai et al., 2009; Mason et al., 2021). However, whether these results are associated with altered REM sleep must be further assessed.

### Other Limitations and Future Steps

Despite the large database used, the number of moderate/severe subjects is relatively low when comparing with the other groups. We have implemented a bootstrap procedure to account for the median of the correlation distributions and minimize the effect of the imbalance. However, future analyses on children with moderate/severe OSA would improve the statistical power of our results. It would also be very interesting to assess our analyses in symptomatic children referred for clinical evaluation. Moreover, there is substantial skepticism as to the validity of AHI and OSA symptomatology or morbidity (Penzel et al., 2015). This AHI limitation may explain why some cognitive scores do not reach significant differences among our OSA severity groups. The cognitive morbidity of OSA is well established (Gozal, 1998; Marcus et al., 2012; Hunter et al., 2016; Tan et al., 2017; Cardoso et al., 2018), and indeed, all our scores exhibit decreasing tendency as OSA worsens. However, the combination of an unclear association between AHI and OSA symptoms and the assessment of a general community-based nonreferral cohort may have resulted in the inclusion of children with AHI ≥ 1 e/h but without any symptoms or morbidity. Another limitation is the specific EEG arrangements we followed. We used the typical EEG channel configuration of sleep studies and the Common Averaged Reference method to minimize the influence of the other channels in each electrode (Cox and Fell, 2020). However, other configurations may lead to different results. On the other hand, we included Nadir_*SpO2*_ in our analyses because it has been observed that the depth of desaturations is associated with increased OSA-related negative consequences in adults (Kulkas et al., 2013; Karhu et al., 2021). However, it would be an interesting future goal to assess other oximetric variables such as oxygen desaturation index or hypoxic burden. Similarly, the inclusion of children’s subjective sleepiness scores in the analysis could complement our findings. Other interesting future goal would be to analyze the EEG recordings by separating REM and NREM sleep stages. In this study, we have shown that OSA-related changes in EEG were evident even without the labor-intensive task of defining REM and NREM sleep. However, this further analysis would help interpret some of our findings. In addition, it could enhance relationships between cognitive scores and specific EEG information in control subjects. Ultimately, another limitation is the age range of the subjects involved in the study. We have conducted several actions to avoid a bias of our results toward age-related natural brain development. First, the age range is not wide (5–9 years). Second, our control and OSA groups are matched in age. Finally, all the correlations used in the study were controlled for age (and sex). However, EEG changes are present in sleep as a consequence of typical development (Gaudreau et al., 2001; Kurth et al., 2010; Gorgoni et al., 2020), which is the reason why our findings should be evaluated in other age ranges.

## Conclusion

Pediatric OSA broadly affects overnight EEG and progressively equates the information of its different spectral bands, regardless of whether it refers to activity or irregularity. Such effects on EEG are coherent with the occurrence of micro and macro sleep disruptions. They also reflect cognitive morbidity, particularly in domains involving language processes, visual–spatial processing, and executive function. Sleep EEG irregularity characterizes a wider range of OSA-related information than the classic activity analysis, which results in more numerous and enhanced robustness in their associations with both physiological and cognitive variables. The results from our correlation network approach were coherent with the previous studies, while expanding the knowledge about the EEG classic spectral bands. Thus, our findings illustrate that the EEG spectrum echoes physiological perturbations during sleep and adverse cognitive consequences of pediatric OSA. It may therefore provide a tool to identify children with OSA who are at increased risk of cognitive deficits, thereby enabling a more personalized approach to its evaluation and management.

## Data Availability Statement

The datasets presented in this article are not readily available because of restrictions from the ethical committee. The data that support the findings of this study are, however, available on reasonable request from the corresponding authors. Requests to access the datasets should be directed to GG-T, gonzalo.gutierrez@gib.tel.uva.es.

## Ethics Statement

The studies involving human participants were reviewed and approved by Ethics Committee of the University of Chicago (protocol # 09-115-B). Written informed consent to participate in this study was provided by the participants’ legal guardian/next of kin.

## Author Contributions

GG-T conceptualized and designed the study, analyzed and interpreted data, drafted the initial manuscript, and reviewed the manuscript. JG-P conceptualized the study, analyzed and interpreted data, and contributed to the manuscript editing and reviewing. LK-G conceptualized the study, recruited and diagnosed the subjects, analyzed the data, and reviewed the manuscript. AM-M, JP and DÁ contributed to the data analysis and interpretation, and reviewed the manuscript. FC contributed to the data analysis and interpretation and critically reviewed the manuscript for important intellectual content. DG and RH conceptualized the study, supervised data collection, conducted the data analysis and interpretation, drafted components of the manuscript, and reviewed the manuscript. All authors contributed to the article and approved the submitted version.

## Conflict of Interest

The authors declare that the research was conducted in the absence of any commercial or financial relationships that could be construed as a potential conflict of interest.

## Publisher’s Note

All claims expressed in this article are solely those of the authors and do not necessarily represent those of their affiliated organizations, or those of the publisher, the editors and the reviewers. Any product that may be evaluated in this article, or claim that may be made by its manufacturer, is not guaranteed or endorsed by the publisher.
