# Supplementary material for: Pediatric Sleep Apnea: The Overnight Electroencephalogram as a Phenotypic Biomarker
Source: Front Neurosci. 2021 Nov 3;15:644697. doi: 10.3389/fnins.2021.644697 (PMC8595944; doi:10.3389/fnins.2021.644697)

# Supplementary Material

# File ‘correlation matrices.xlsx’

**Figure SM1. Standardized centrality measures (strength, closeness, and betweenness) for the control EEG activity correlation network**. Nodes are sorted in ascending order of *strength*.

**Figure SM2. Standardized centrality measures (strength, closeness, and betweenness) for the mild EEG activity correlation network**. Nodes are sorted in ascending order of *strength*.

**Figure SM3. Standardized centrality measures (strength, closeness, and betweenness) for the moderate/severe OSAS EEG activity correlation network**. Nodes are sorted in ascending order of *strength*.

**Figure SM4. Standardized centrality measures (strength, closeness, and betweenness) for the control EEG irregularity correlation network**. Nodes are sorted in ascending order of *strength*.

**Figure SM5. Standardized centrality measures (strength, closeness, and betweenness) for the mild OSAS EEG irregularity correlation network**. Nodes are sorted in ascending order of *strength*.

**Figure SM6. Standardized centrality measures (strength, closeness, and betweenness) for the moderate/severe OSAS EEG irregularity correlation network.** Nodes are sorted in ascending order of *strength*.


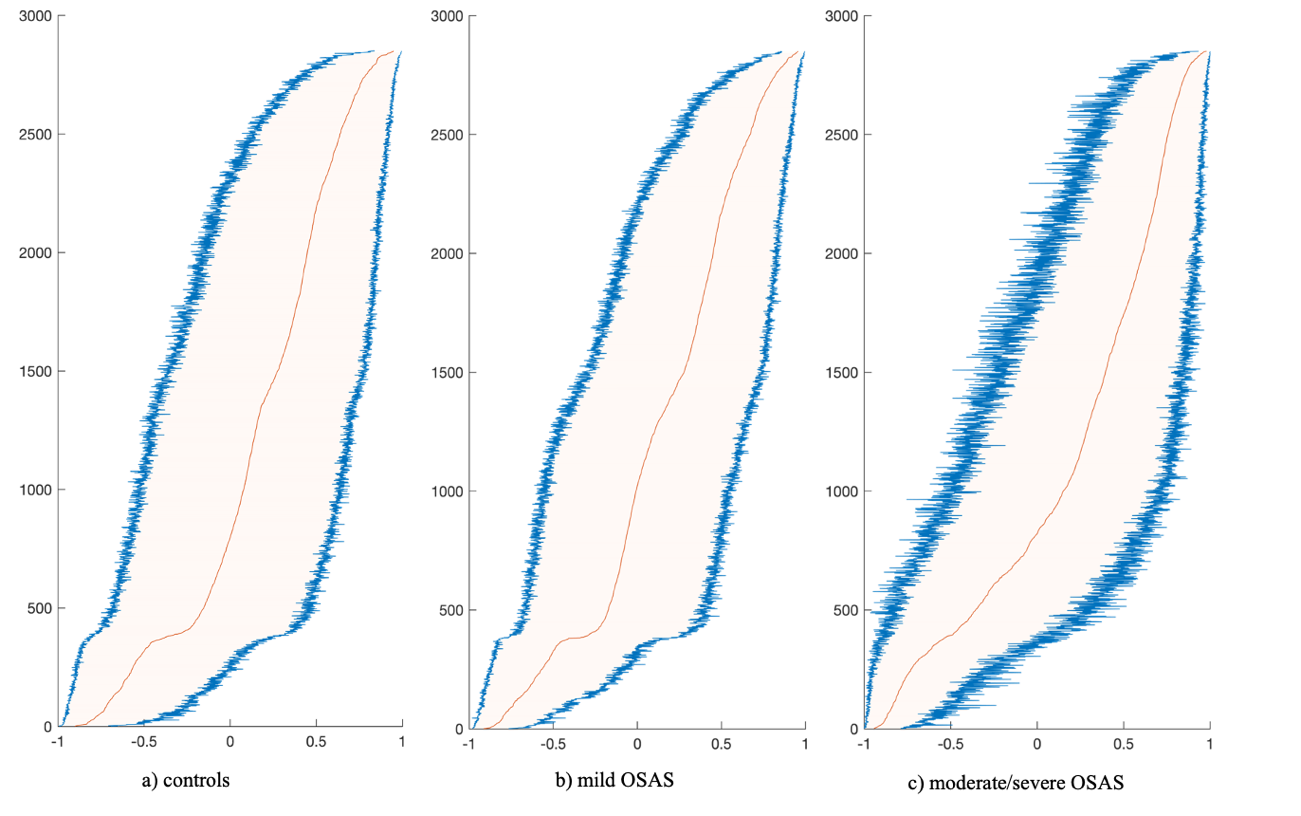
**Figure SM7. Bootstrapped stability of each edge of the EEG activity networks for a) controls, b) mild OSAS, and c) moderate/severe OSAS**. Blue limits are 2.5 and 97.5 percentiles of correlation distribution for each edge. Brown dots are the corresponding median values

**Figure SM8. Bootstrapped stability of each edge of the EEG irregularity networks for a) controls, b) mild OSAS, and c) moderate/severe OSAS.** Blue limits are 2.5 and 97.5 percentiles of correlation distribution for each edge. Brown dots are the corresponding median values


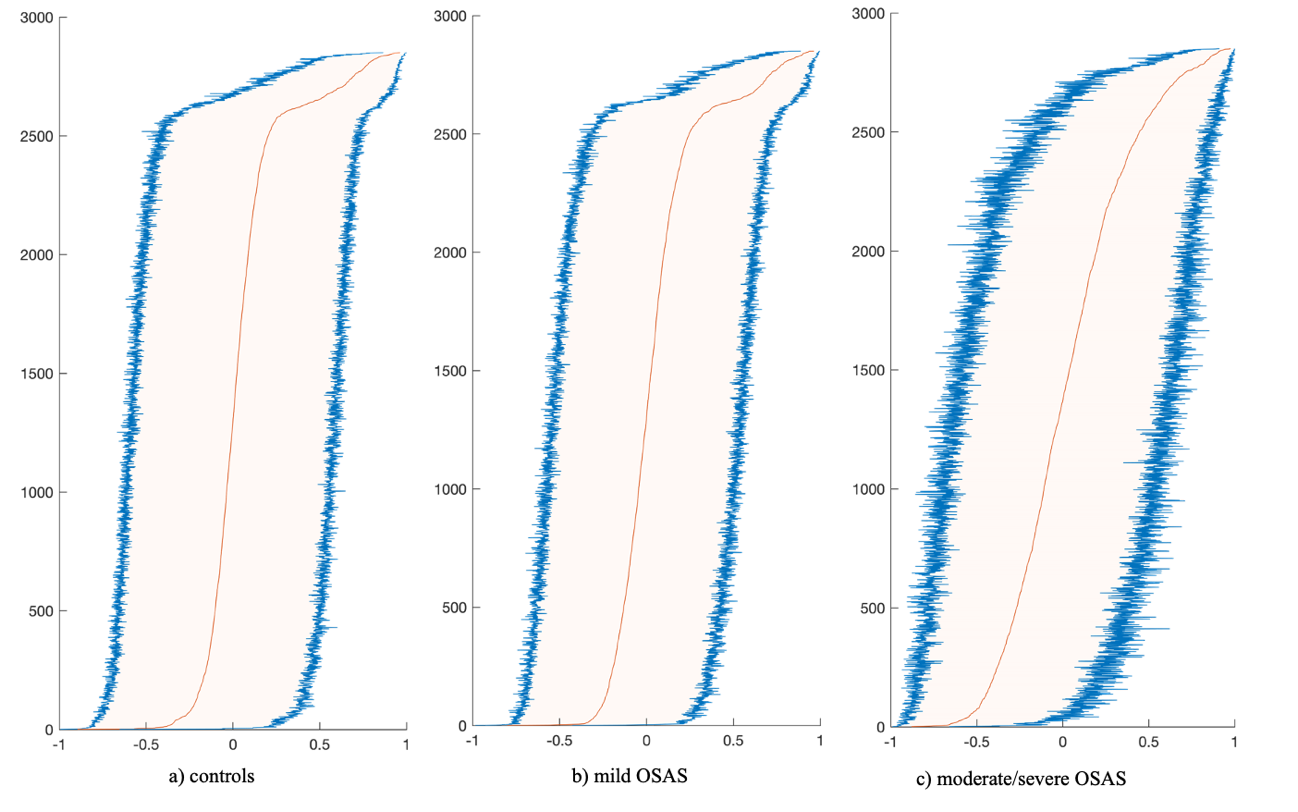

Supplement: Supplementary file 1 [file Data_Sheet_1.docx]
